# ^18^F-Fluoroethylcholine PET/CT Radiomic Analysis for Newly Diagnosed Prostate Cancer Patients: A Monocentric Study

**DOI:** 10.3390/ijms23169120

**Published:** 2022-08-14

**Authors:** Daniele Antonio Pizzuto, Elizabeth Katherine Anna Triumbari, David Morland, Luca Boldrini, Roberto Gatta, Giorgio Treglia, Riccardo Bientinesi, Marco De Summa, Marina De Risi, Carmelo Caldarella, Eros Scarciglia, Angelo Totaro, Salvatore Annunziata

**Affiliations:** 1 Nuclear Medicine Unit, TracerGLab, Department of Radiology, Radiotherapy and Hematology, Fondazione Policlinico Universitario A. Gemelli IRCCS, 00168 Rome, Italy; 2 Nuclear Medicine, Institut Godinot, 51100 Reims, France; 3 CReSTIC (Centre de Recherche en Sciences et Technologies de l’Information et de la Communication), EA 3804, Université de Reims Champagne-Ardenne, 51100 Reims, France; 4 Radiotherapy Unit, Radiomics, Department of Radiology, Radiotherapy and Hematology, Fondazione Policlinico Universitario A. Gemelli IRCCS, 00168 Rome, Italy; 5 Department of Clinical and Experimental Sciences, University of Brescia, 25121 Brescia, Italy; 6 Clinic of Nuclear Medicine, Imaging Institute of Southern Switzerland, Ente Ospedaliero Cantonale, 6500 Bellinzona, Switzerland; 7 Faculty of Biology and Medicine, University of Lausanne, 1015 Lausanne, Switzerland; 8 Faculty of Biomedical Sciences, Università della Svizzera italiana, 6900 Lugano, Switzerland; 9 Clinical Urology Division, Department of Medical and Surgical Sciences, Fondazione Policlinico Universitario A. Gemelli IRCCS, 00168 Rome, Italy; 10 Medipass S.p.a., Servizio Integrativo PET-TC Radiofarmacia, Fondazione Policlinico Universitario A. Gemelli IRCCS, 00168 Rome, Italy

**Keywords:** radiomics, PET, Choline, prostate cancer, staging, nuclear medicine

## Abstract

Aim: The aim of this study is to assess whether there are some correlations between radiomics and baseline clinical-biological data of prostate cancer (PC) patients using Fluorine-18 Fluoroethylcholine (^18^F-FECh) PET/CT. Methods: Digital rectal examination results (DRE), Prostate-Specific Antigen (PSA) serum levels, and bioptical-Gleason Score (GS) were retrospectively collected in newly diagnosed PC patients and considered as outcomes of PC. Thereafter, Volumes of interest (VOI) encompassing the prostate of each patient were drawn to extract conventional and radiomic PET features. Radiomic bivariate models were set up using the most statistically relevant features and then trained/tested with a cross-fold validation test. The best bivariate models were expressed by mean and standard deviation to the normal area under the receiver operating characteristic curves (mAUC, sdAUC). Results: Semiquantitative and radiomic analyses were performed on 67 consecutive patients. tSUVmean and tSkewness were significant DRE predictors at univariate analysis (OR 1.52 [1.01; 2.29], *p* = 0.047; OR 0.21 [0.07; 0.65], *p* = 0.007, respectively); moreover, tKurtosis was an independent DRE predictor at multivariate analysis (OR 0.64 [0.42; 0.96], *p* = 0.03) Among the most relevant bivariate models, szm_2.5D.z.entr + cm.clust.tend was a predictor of PSA levels (mAUC 0.83 ± 0.19); stat.kurt + stat.entropy predicted DRE (mAUC 0.79 ± 0.10); cm.info.corr.1 + szm_2.5D.szhge predicted GS (mAUC 0.78 ± 0.16). Conclusions: tSUVmean, tSkewness, and tKurtosis were predictors of DRE results only, while none of the PET parameters predicted PSA or GS significantly; ^18^F-FECh PET/CT radiomic models should be tested in larger cohort studies of newly diagnosed PC patients.

## 1. Introduction

Prostate cancer (PC) is the most frequent type of cancer in men worldwide. PC patients may be treated with radical prostatectomy or radiotherapy as primary treatments, but they may develop disease recurrence. Although the 10-year survival rate of PC patients is approximately 90% [1], 20–40% of patients will relapse [2,3,4]. Risk classification of patients with PC mainly relies on clinical examination, biochemical data, and Gleason Score (GS) [5], the latter being restricted to the biopsied portion of the prostate [6].

Positron Emission Tomography (PET) combined with Computed Tomography (PET/CT) or Magnetic Resonance (PET/MR) using several radiopharmaceuticals are new-generation imaging techniques allowing a more accurate and precise staging and restaging of PC compared to conventional imaging methods due to the advantage of combining functional and morphological information [7]. Radiolabelled choline is a radiopharmaceutical used in PET imaging as a marker of cell membrane synthesis, which is increased in PC. In particular, Fluorine-18 Fluoroethylcholine (^18^F-FECh) PET/CT is a well-established non-invasive tool for restaging and staging/restaging settings of PC, with a reported patient-based overall sensitivity of 93% and a specificity of 83% [8,9].

Radiomics is a high-throughput approach that translates medical images into minable data by extracting many quantitative features describing the intensity, shape, and heterogeneity of targeted lesions. Radiomic models could indirectly be an expression of tumor biology behavior and could be considered an additional support, together with clinical and histological data, to help the clinicians in the diagnostic iter [10]. Radiomics applied to ^18^F-FECh PET/CT was recently shown to predict PC outcome at follow-up [11]. However, the association between ^18^F-FECh PET/CT radiomic features and initial tumor characteristics that may underlie these results has not been well described so far. Our study aimed at correlating the semiquantitative and radiomic parameters extracted from the prostate showing high ^18^F-FECh uptake with baseline clinical assessment and histological data in patients undergoing PET/CT scans for initial staging.

## 2. Results

Data from 67 patients were retrospectively collected. Mean Prostate-Specific Antigen (PSA) serum values were 15.0 ± 13.0 ng/mL. Results from Digital Rectal Examination (DRE) were available in 63/67 (94.0%) patients; 26/63 (41.3%) were suspicious for PC, 37/63 (58.7%) were negative. The Gleason Score (GS) was ≤7 in 29/67 patients (43.3%). Table 1 shows the demographic characteristics of the cohort.

At univariate analysis, none of the conventional PET parameters was significantly predictive of PSA serum values higher than PSA median value (9.3 ng/mL) or GS > 7. tSUVmean (mean concentration values of 18F-FeCh counts within the prostate, normalized by the patient’s body weight), tSkewness and tKurtosis (histogram features, which describe the simmetricity and shape of 18F-FeCh counts in the prostate, respectively) were significant predictors of DRE results at univariate analysis (OR 1.52 [1.01; 2.29], *p* = 0.047; OR 0.21 [0.07; 0.65], *p* = 0.007; OR 0.60 [0.39; 0.91], *p* = 0.018, respectively). Among them, only tKurtosis was shown to be an independent predictive parameter in the multivariate analysis (OR 0.64 [0.42; 0.96], *p* = 0.03) (Table 2).

The best models for each outcome were: szm_2.5D.z. entr + cm.clust.tend for PSA levels (mAUC 0.83 ± 0.19); stat.kurt + stat.entropy for DRE (mAUC 0.79 ± 0.10); cm.info. corr.1 + szm_2.5D. szhge for GS (mAUC 0.78 ± 0.16) (Table 3). However, none of these models reached statistical significance.

## 3. Discussion

### 3.1. Conventional Semiquantitative PET Parameters

This single-center retrospective study explored the association of semiquantitative PET parameters and radiomic features derived from primary tumor ^18^F-FECh uptake with baseline clinical-biological data of patients with newly diagnosed PC.

None of the conventional PET parameters was a predictor of PSA values higher than PSA median value (9.3 ng/mL) or GS > 7. Volume, as explored by tMTV, was not associated with DRE positivity, while prostatic tKurtosis was, even at multivariate analysis. Our main hypothesis is that kurtosis, which represents a histogram feature and describes the shape of the distribution of ^18^F-FECh values within the prostate affected by the tumor, conveys some sense of firmness or irregularity of the prostatic gland. Although not clinically relevant, this result may improve our understanding of the relationship between radiomic factors and the physical and biological properties of prostatic pathological tissues.

The role of semiquantitative ^18^F-FECh PET/CT parameters is controversial in initial PC staging. Zanoni et al. showed a significant correlation between TBR and the International Society of Urological Pathology Score (derived from GS), using the liver as the reference organ [12]. These data are discrepant with our results, at least partially owing to the fact that we used the spleen as the reference organ. This choice was originally justified by the prevalence of metastases in the liver (about 10% [13], while they are very unusual in the spleen) and their detection difficulty on ^18^F -FECh PET/CT [14].

Semiquantitative PET parameters were revealed to be useful for significantly discriminate malignant vs. non-malignant lesions [12]. Schaefferkotter et al. showed that SUV could be a strong predictor of aggressive disease in the prostate (GS ≥ 4 + 3) [15]. Conversely, semiquantitative analysis would not be able to discriminate among less-aggressive PC or benign conditions, such as foci of prostatitis and benign prostatic hyperplasia, because all of them are characterized by high tracer uptake due to the increased choline transport and overexpression of choline kinase in all these conditions [16].

### 3.2. Advanced Radiomic Features

**Figure 1:**
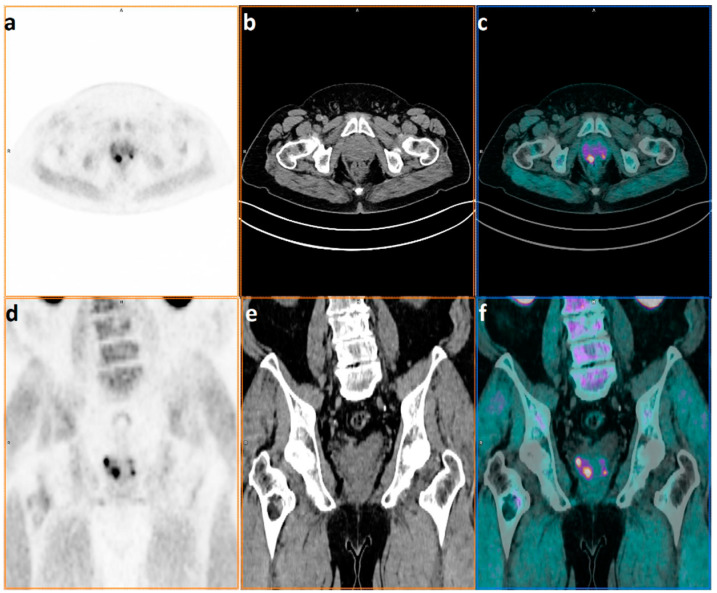
Patient with diagnosis of prostate cancer referred to ^18^F-FECh PET/CT for staging in May 2021; Gleason Score 9 = 4 + 5 and PSA value at diagnosis of 9.74 ng/mL. PET only, co-registered CT only, and hybrid PET/CT images in axial (**a**–**c**) and coronal plans (**d**–**f**) showing a multifocal pattern with four areas of focal uptake in the prostate, of which the most evident is placed in the peripheral zone of the right lobe.

**Figure 2:**
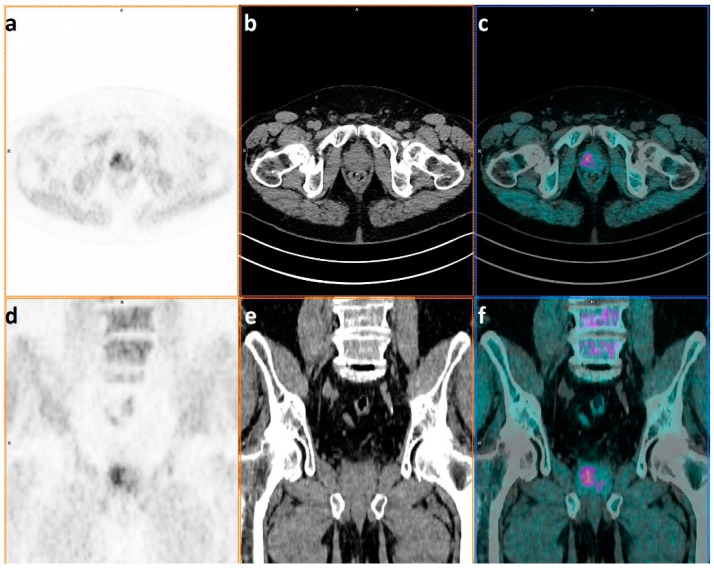
Patient with diagnosis of prostate cancer referred to ^18^F-FECh PET/CT for staging in February 2021; Gleason Score 7 = 4 + 3 and PSA value at diagnosis of 7.62 ng/mL. PET only, co-registered CT only, and hybrid PET/CT images in axial (**a**–**c**) and coronal plans (**d**–**f**) showing a diffuse pattern of ^18^F-FECh uptake, mostly involving the right lobe of the prostate.

The analysis of advanced radiomic parameters provided richer results, as a bivariate model with an accuracy of about 80% was found for each of the 3 outcomes: szm_2.5D.z. entr + cm.clust.tend for PSA levels (mAUC 0.83 ± 0.19), stat.kurt + stat.entropy for DRE (mAUC 0.79 ± 0.10), and cm.info. corr.1 + szm_2.5D.szhge for GS (mAUC 0.78 ± 0.16). The physical meaning of these features is less easily conceptualized, however, with several measures of heterogeneity (entropy). The association of textural parameters and GS opens interesting perspectives, as PET provides information on the whole prostate. The heterogeneous texture might not concern only the high uni- or multifocal ^18^F-FECh uptake areas but could involve the entire gland (Figure 1 and Figure 2). In line with this concept, Tu et al. assessed the predictive role of 3 radiomic zones within the prostate (zone 1 corresponding to the high metabolic volume with 40–100% of SUVmax value; zone 2, corresponding to the peripheral tumor zone with 30–40% SUVmax value; zone 3, corresponding to the extended peripheral tumor zone in which the entire prostate organ and zone 2 is included) for risk classification of PC patients referred to Choline PET/MR. The authors found that the zone 1 radiomics could be superior to PSA-based evaluation for patients’ risk classification; moreover, both zone 2 and zone 3 were shown to be superior to TNM-based assessment for risk classification [17]. In our cohort, neither conventional PET parameters nor advanced radiomic models were able to discriminate significantly between low and high GS tumors. Our findings were different from the results drawn by Zamboglou et al., which showed the feasibility of using ^68^Ga-PSMA radiomic features to discriminate GS 7 and GS 8 lesions [18]. Such discrepancy could at least partially rely on the different radiotracers and different scanners. Despite the role of radiomic models applied to the ^18^F-FECh PET/CT images in PC patients that are emerging with the aim to provide support for clinical decisions in this setting in the era of personalized medicine, risk classification is still based on biopsy-based GS, which is not always representative of the whole tumor and may lead to side effects, such as hematuria and/or hematospermia, in addition to the trend of under- or over-grading primary PC [1]. Hence, imaging is still used for displaying suspected lesions and/or for biopsy guidance [19,20].

However, it should be kept in mind that ^18^F-FECh PET/CT remains a well-established second-line diagnostic tool for clinical management of PC patients, with PSA values representing the main weapon for screening men of a certain age or with symptoms suspected of PC, such as dysuria. Furthermore, collecting a small amount of peripheral blood to assess PSA serum values is indisputably more convenient and feasible than the ^18^F-FECh PET/CT procedure in terms of radiation exposure or medical expense. In summary, ^18^F-FECh PET/CT represents a support, not an alternative tool for clinical baseline decision-making in PC patients, solidly based on GS and PSA values.

### 3.3. Limitations and Solutions

The main limitation of our study is its retrospective nature and, in particular, the heterogeneity of the PET/CT scanners in terms of hardware (digital vs. non-digital PET) and software (reconstruction, time of flight). Nevertheless, cross-fold validation ensured that the identified parameters were at least somewhat robust across the three scanners.

The number of included patients is overall low, but enough to derive significant models. Confirmatory studies should also be conducted to provide an external validation cohort for the confirmation of the observed results.

This work was confined to the study of the association between PET and clinical-biological variables without any prognostic aim. Furthermore, our results are limited as they take into account the lack of definitive histopathological results of the whole prostate specimen.

Other studies may be needed to explore survival outcomes.

## 4. Materials and Methods

Patients with biopsy-confirmed PC who underwent a staging ^18^F-FECh PET/CT in the PET/CT Center of Fondazione Policlinico Universitario A. Gemelli IRCCS (Rome, Italy) between June 2018 and March 2021 were retrospectively recruited for this single-center study. The following parameters were collected: age, results of DRE, Prostate-Specific Antigen (PSA) levels, and biopsy-based Gleason Score (GS). The retrospective use of data from clinical routine was performed according to institutional rules. The study was approved by the local Ethical Committee, ID number 5177. All procedures performed were in accordance with the ethical standards defined by the 1964 Helsinki Declaration and its later amendments.

### 4.1. Acquisition Protocol and Image Analysis

After biopsy-based confirmation of PC, all patients underwent ^18^F-FECh PET/CT for staging purposes. Philips Gemini XL, Siemens Biograph mCT, and Siemens Biograph Vision V600 scanners were used. After a first non-contrast enhanced pelvic CT, 4MBq/kg of ^18^F-FECh were intravenously administered to the patient in a supine position and immediately followed by a 5-min early PET acquisition of the pelvic region. A whole-body skull-base-to-thigh PET/CT was performed about 60 min after ^18^F-FECh administration. Images derived from Philips GEMINI XL were reconstructed by the LOR method, while images from both Siemens Biograph mCT and Siemens Biograph Vision V600 scanners were reconstructed by True-X TOF (ultraHD PET) iterative reconstruction algorithms (Table 4).

A Volume of interest (VOI) was manually drawn for each patient to contour the prostate gland showing suspicious ^18^F-FECh uptake by applying a gradient-based threshold using a PET segmentation tool (LesionID, version 7.0.5 of MIM Encore Software Inc., Cleveland, OH, USA) and manual corrections were performed, when necessary, to avoid the inclusion in VOIs of spill-over areas due to ^18^F-FECh accumulation in the bladder.

Conventional semiquantitative PET parameters were extracted from each VOI (t: tumor):

- Maximum and mean Standardized Uptake Value, which indirectly estimates the maximum and mean values of 18F-FeCh concentration within the VOI by the normalization with the patient’s body weight (tSUVmax and tSUVmean, respectively);
- Metabolic Tumor Volume (tMTV), which represents the volume involving all the ^18^F-FECh counts with at least 40% of SUVmax value;
- Total Lesion Activity (tTLA as expression of tSUVmean × tMTV);
- First-order radiomic features, such as tSkewness and tKurtosis, which describe the asimmetricity and the shape of distribution of ^18^F-FECh values within the VOI, respectively.

Additionally, a 2-cm spherical VOI was drawn on the spleen (Sp) to extract spleen SUVmean (SpSUVmean) and its standard deviation (SUVsd) as estimates of background intensity and noise, respectively.

Tumor-to-background ratio (TBR) and Signal-to-noise ratio (SNR) were calculated as follows: tSUVmax/SpSUVmean and tSUVmax/SUVsd, respectively. These ratios were introduced and included in the analysis to normalize the variability caused by the different scanners.

Advanced radiomic features were extracted from each prostatic VOI using the PET/CT module of an open-source IBSI-compliant platform (Moddicom) [21].

### 4.2. Statistical Analysis

Quantitative variables were described by mean and standard deviation, while qualitative variables by number and percentage.

A first analysis was performed considering the tumor’s conventional PET parameters and first-order radiomic features (tSUVmax, tSUVmean, tMTV, tTLA, TBR, SNR, tSkewness, tKurtosis). For each binarized outcome (dichotomized PSA using median, dichotomized GS (6-7 vs. 8-9), and dichotomized DRE results suspicious vs. negative), logistic regression was performed to assess the predictive power of PET variables. Results were presented as odd ratios (OR) and corresponding 95% confidence intervals (CI). A multivariable model selection was performed among significant variables using Akaike’s information criterion maximization.

The Mann–Whitney U test was used to evaluate the predictive role of advanced radiomic features with respect to the same binarized outcomes (PSA, DRE, GS). Radiomic bivariate models were built using the most relevant features (with *p* < 0.05 at univariate analysis) through logistic regression and trained/tested with a cross-fold validation test (80% vs. 20%, 10 repetitions). The best bivariate models were selected based on receiver operating characteristic (ROC) curves, mean area under the ROC curves (mAUC), and standard deviation (sdAUC).

## 5. Conclusions

Our study showed that tSUVmean only among semiquantitative parameters, tSkewness and tKurtosis as first order radiomic features were predictors of DRE results, while none of the semiquantitative parameters were able to be significantly correlated to the main clinical/histological parameters in PC patients, such as low/high PSA values and low and high-risk GS in PC patients referring ^18^F-FECh PET/CT for primary staging. Larger prospective validation cohorts are recommended to explore if validated ^18^F-FECh PET/CT radiomic models could represent an additional tool in the clinical management of newly diagnosed PC patients in the era of personalized medicine.

## Figures and Tables

**Table 1:** Descriptive analysis of patients’ characteristics.

	*n* = 67
Age in years (mean ± sd)	70.1 ± 7.1
PSA in ng/mL (mean ± sd)	15.0 ± 13.0
Digital Rectal Examination (*n*, %)	
Suspicious	26 (41.3%)
Negative	37 (58.7%)
Missing data	4 (6.0%)
Gleason score (*n*, %)	
6 = 3 + 3	1 (1.5%)
7 = 3 + 4	8 (11.9%)
7 = 4 + 3	20 (29.9%)
8 = 3 + 5	1 (1.5%)
8 = 4 + 4	19 (28.3%)
9 = 4 + 5	17 (25.4%)
9 = 5 + 4	1 (1.5%)
PET-CT system (*n*, %)	
Gemini XL	39 (58.2%)
Biograph mCT	22 (32.8%)
Biograph Vision V600	6 (9.0%)
PET parameters (mean ± sd)	
tSUVmax	10.4 ± 4.4
tSUVmean	3.9 ± 1.5
tMTV (mL)	16.0 ± 11.7
tTLA	60.6 ± 51.6
tSkewness	1.1 ± 0.7
tKurtosis	2.2 ± 3.7
TBR	2.7 ± 1.1
SNR	15.3 ± 6.4

PSA, prostate-specific-antigen; sd, standard deviation; SNR, Signal-to-noise ratio; tMTV, metabolic tumor volume of the lesion; t TLA, total lesion activity of the lesion; TBR, Tumor-to-background ratio.

**Table 2:** Univariate and multivariate analysis performed for PSA, Gleason Score, and Digital Rectal Examination results prediction. If *p*-value is statistically significant (<0.05, marked by an asterisk), OR and 95% CI are presented. Only *p*-value is reported if not significant. PSA, prostate-specific-antigen; SNR, Signal-to-noise ratio; tMTV, metabolic tumor volume of the lesion; tTLA, total lesion activity of the lesion; TBR, Tumor-to-background ratio.

	PSA > Median Value (9.3 ng/mL)	GS 6-7 vs. 8-9	Digital Rectal Examination Results	
	Univariate	Univariate	Univariate	Multivariate
tSUVmax	0.285	0.664	*p* = 0.701	-
tSUVmean	0.074	0.306	*p* = 0.047 * 1.52 [1.01; 2.29]	*p* = 0.13
tMTV (mL)	0.210	0.447	*p* = 0.867	-
tTLA	0.195	0.954	*p* = 0.400	-
tSkewness	0.345	0.188	*p* = 0.007 * 0.21 [0.07; 0.65]	Rejected
tKurtosis	0.196	0.135	*p* = 0.018 * 0.60 [0.39; 0.91]	*p* = 0.03 * 0.64 [0.42; 0.96]
TBR	0.319	0.378	*p* = 0.313	-
SNR	0.091	0.723	*p* = 0.752	-

PSA, prostate-specific-antigen; SNR, Signal-to-noise ratio; tMTV, metabolic tumor volume of the lesion; t TLA, total lesion activity of the lesion; TBR, Tumor-to-background ratio.

**Table 3:** Bivariate analysis among considered clinical outcomes, i.e., PSA, digital rectal examination, and Gleason Score and most relevant radiomic features at univariate analysis.

	cov.1.cov.1	cov.2.cov.2	*p*.Value.1	sd.*p*.Value.1	*p*.Value.2	sd.*p*.Value.2	AUC	sd.AUC
PSA	szm_2.5D.z.entr	cm.clust.tend	0.079	0.035	0.132	0.075	0.829	0.195
DRE	stat.kurt	Stat.entropy	0.136	0.048	0.371	0.145	0.787	0.097
GS	cm.info.corr.1	rlm.hgre	0.410	0.214	0.219	0.114	0.812	0.118

AUC: Area under the Curve; DRE: digital rectal examination; GS: Gleason Score; PSA: prostate-specific antigen; sd: standard deviation.

**Table 4:** Acquisition modalities and image reconstruction algorithms for each scanner.

	Philips Gemini XL	Siemens Biograph mCT	Siemens Biograph Vision V600
Low dose CT scan	120 kV, 40–50 mAs	120 kV, 40–50 mAs	120 kV, 40–50 mAs
Acquisition time and modality	2 min per bed	2 min per bed	PET continuous bed motion: 1–2 mm/sec
Image Reconstruction	LOR RAMLA reconstruction without PSF and TOF (3 iterations and 33 subsets, voxel size: 4 × 4 × 4 mm^3^)	UltraHD-PET: line-of- response row-action maximum likelihood algorithm 3D OSEM reconstruction + PSF modeling + TOF (2 iterations, 21 subsets, voxel size: 3.2 × 3.2 × 5 mm^3^),	UltraHD-PET: line-of- response row-action maximum likelihood algorithm 3D OSEM reconstruction + PSF modeling + TOF (4 iterations and 5 subsets, voxel size of 1.8 × 1.8 × 5 mm^3^),

CT, Computed Tomography; HD, High Definition; LOR, Line of Response; OSEM, Ordered Subset Expectation Maximization; PSF, Point Spread Function; RAMLA; Row-Action Maximum Likelihood Algorithm; TOF, Time Of Flight.

## Data Availability

Patient data were collected and stored by D.A.P and shareable if requested.
